# Visualization of the chemical defense molecule formoside binding to sensory structures in a model fish predator

**DOI:** 10.1242/jeb.246246

**Published:** 2023-12-21

**Authors:** Samantha J. Mascuch, Bhuwan Khatri Chhetri, Nazia Mojib, Julia Kubanek

**Affiliations:** ^1^ School of Biological Sciences, Georgia Institute of Technology, Atlanta, GA 30332, USA; ^2^ School of Chemistry & Biochemistry, Georgia Institute of Technology, Atlanta, GA 30332, USA; ^3^ Department of Biology, Spelman College, Atlanta, GA 30314, USA; ^4^ Center for Microbial Dynamics and Infection, Parker H. Petit Institute for Bioengineering and Bioscience, Georgia Institute of Technology, Atlanta, GA 30332, USA

**Keywords:** Chemoreception, Zebrafish, *Danio rerio*

## Abstract

Sensory perception of chemical threats coming from an organism's environment relies on the coordination of numerous receptors and cell types. In many cases, the physiological processes responsible for driving behavioral responses to chemical cues are poorly understood. Here, we investigated the physiological response of fish to an unpalatable compound, formoside, which is employed as a chemical defense by marine sponges. Construction of fluorescent probe derivatives of formoside allowed visualization of this chemical defense molecule *in vivo*, interacting with the cells and tissues of the early larvae of a model predator, the zebrafish (*Danio rerio*). This revealed the precise chemosensory structures targeted by formoside to be in the taste buds and olfactory epithelium of developing zebrafish. Mechanosensory neuromasts were also targeted. This study supports the involvement of a previously identified co-receptor in detection of the chemical defense and provides a springboard for the long-term goal of identification of the cellular receptor of formoside. Extension of this approach to other predators and chemical defenses may provide insight into common mechanisms of chemoreception by predators as well as common strategies of chemical defense employed by prey.

## Teaser

**Summary:** A chemical defense molecule, formoside, is localized to taste buds, olfactory tissue and neuromasts of larval zebrafish, used as a model fish predator.

## INTRODUCTION

Olfaction and gustation in fishes are essential to their ability to determine the palatability of food items, recognize threats, distinguish kin, coordinate mating and navigate their environment. Cellular receptor types responsible for molecular recognition of odorants and taste molecules can be G protein-coupled receptors (GPCRs), ion channels or guanylate cyclases, with receptors present on both olfactory sensory neurons (OSNs) and taste receptor cells (Korsching, 2020). Whereas OSNs are restricted to the olfactory epithelium that lines the olfactory organ, taste buds can be located orally on the palate or lips, or may be extraoral on the gills, barbels, fins and trunk. Extraoral taste buds are characterized by greater sensitivity than oral taste buds, consistent with their role in sensing food at a distance; oral taste buds are responsible for the ultimate determination of palatability (Kasumyan, 1997).

Some ligands for chemosensation have been identified in fishes, although in many cases specific receptor identity is unknown (Oike et al., 2007; Ahuja and Korsching, 2014; Wakisaka et al., 2017; Cong et al., 2019). This is especially true in the study of the naturally occurring chemical defenses of aquatic prey such as sponges, corals, mollusks, macroalgae and benthic cyanobacteria. Chemicals that these organisms use to protect themselves from predation may be unpalatable and/or toxic (Pawlik, 1993; Pawlik et al., 1995). Assays for evaluating the feeding deterrence of these chemicals towards fishes are generally behavioral in nature (e.g. based on observation of fish acceptance or rejection of chemically laced food items) (Hay et al., 1998). Although this approach is ecologically relevant, it does not lead to the deciphering of the underlying molecular physiology driving the behavioral response.

Formoside is a specialized metabolite of the triterpene glycoside family, found in tissues of the Caribbean sponge *Erylus formosus*, that deters predation by numerous species of reef fishes (Kubanek et al., 2000). Feeding deterrence in response to formoside has also been demonstrated in the model zebrafish (*Danio rerio*) (Cohen et al., 2008). Relative to other marine chemical defenses, the signaling machinery responsible for formoside feeding deterrence (and that of triterpene glycosides in general) is well studied. The chemical signaling pathway has been functionally reconstituted in *Xenopus laevis* oocytes, and a putative co-receptor, RAMP-like triterpene glycoside receptor (RL-TGR), that functions in conjunction with a GPCR to sense formoside has been characterized in, and directly detected from, the model zebrafish (Cohen et al., 2008, 2010).

RL-TGR is classified as a receptor-activity modifying protein (RAMP). As small proteins that consist of a single transmembrane domain, an extracellular N-terminal domain and a short C-terminal intracellular domain, RAMPs act as chaperones to modify the activity of a GPCR through alteration of its ligand selectivity, signaling or trafficking (Hay and Pioszak, 2016). RAMPs therefore expand the detection capabilities of a fixed number of GPCRs within a sensory system. RAMP involvement in fish aversion to prey chemical defenses is a newly appreciated mechanism within aquatic sensory physiology. The identity of the GPCR with which RL-TGR acts is unknown. However, expression of RL-TGR in zebrafish olfactory epithelium and taste buds supports its involvement in chemical sensing (Mojib et al., 2017).

Although a chemical signaling pathway reconstituted in *X. laevis* oocyctes and consisting of the cystic fibrosis transmembrane conductance regulator (CFTR) chloride channel, beta-2 adrenergic receptor (β_2_AR, a GPCR) and a zebrafish cDNA clone encoding RL-TGR was responsive to formoside in electrophysiology assays, direct interaction of formoside with RL-TGR or other receptors in fishes has not been documented. Therefore, we set out to advance understanding of fish triterpene glycoside sensation by directly observing the interaction of formoside with zebrafish receptors, cells and tissues using fluorescent probes and immunofluorescence microscopy. We hypothesized that formoside localizes to zebrafish tissue surfaces in areas rich in chemosensory cells and would overlap with regions of RL-TGR expression.

## MATERIALS AND METHODS

### Chemicals

Formoside used in probe construction was purified according to previously published methods and its molecular structure and purity were confirmed by nuclear magnetic resonance (NMR) spectroscopy and mass spectrometry (MS) as previously described (Kubanek et al., 2000, 2001). BDP FL-PEG4-amine TFA salt was purchased from BroadPharm (San Diego, CA, USA). Cyclododecane carboxylic acid used in construction of the control probes was purchased from Sigma-Aldrich (St Louis, MO, USA). 2-(3-But-3-yn-1-yl)-3H-diazirin-3-yl) ethan-1-amine used in construction of biotinylated photoaffinity probes was purchased from AstaTech Inc. (Bristol, PA, USA). Biotin-PEG11-azide used in construction of biotinylated photoaffinity probes was purchased from BroadPharm. Other materials and reagents used for peptide coupling were previously obtained from commercial sources and were used without further purification. Ultrapure dimethyl sulfoxide (DMSO) used to solubilize chemical probes for use in bioassays was purchased from Fisher Scientific (Pittsburgh, PA, USA). Fluorescein streptavidin used to visualize biotinylated probes via confocal microscopy was purchased from Vector Laboratories (Burlingame, CA, USA). Synthetic schemes for probe construction are shown in Figs S1 and S2. ^1^H NMR and mass spectral data are available upon request from the corresponding author.

### Construction of BDP FL-PEG4-formoside (=formoside-BODIPY) probe via peptide coupling

Formoside (6.0 mg, 0.0060 mmol) was dissolved in 1 ml dimethylformamide (DMF) and stirred under ambient conditions. HATU (1.5 equiv., 3.4 mg) and DIPEA (2 equiv., 2.0 μl) were added in succession and the mixture was stirred for 15 min prior to the addition of BDP FL-PEG4-amine (1.5 equiv., 4.6 mg). After 16 h, the progress of the reaction was evaluated by thin layer chromatography (TLC) on aluminum-backed silica plates eluting with 8.8:2.2:1.6 ethyl acetate:methanol:water. TLC spots were evaluated with UV light at 254 and 365 nm prior to being charred with 5% sulfuric acid in ethanol.

### Construction of BDP FL-PEG4-cyclododecane (=cyclododecane-BODIPY) probe via peptide coupling

Cyclododecanecarboxylic acid (1.1 mg, 0.0053 mmol) was dissolved in 1 ml DMF and stirred under ambient conditions. HATU (1.5 equiv., 3.0 mg) and DIPEA (2 equiv., 2.0 μl) were added in succession and the mixture was stirred for 15 min prior to the addition of BDP FL-PEG4-amine (1.5 equiv., 4.0 mg). After 16 h, the progress of the reaction was evaluated as described above.

### Peptide coupling of formoside and amine-diazirine-alkyne

Formoside (11 mg, 0.011 mmol) was introduced into a clean amber vial. HCTU (1.5 equiv., 0.016 mmol) was added in DMF and stirred for 20 min, then 2-(3-but-3-yn-1-yl)-3H-diazirin-3-yl)ethan-1-amine (2 equiv., 0.021 mmol) was added and the reaction was stirred for an additional 30 min. DIPEA (3 equiv., 0.0032 mmol, 5.6 μl) was added to the reaction. The upper layer was purged with argon and sealed and the reaction was stirred at room temperature for 16 h. The progress of the reaction was evaluated as above.

### Construction of formoside-diazirine-biotin probe via click chemistry

Formoside-diazirine-alkyne was joined to biotin-PEG11-azide via copper(I)-catalyzed alkyne-azide cycloaddition (CuAAC). Formoside-diazirine-alkyne (7.7 mg, 0.066 mmol) was resuspended in 2.85 ml 1:1 tert-butanol:H_2_O for a final concentration of 2.3 mmol l^−1^ alkyne. Biotin-PEG11-azide (9.65 mmol l^−1^, 1.3 ml) was added to the alkyne. A freshly prepared, premixed solution of THPTA ligand (33.3 mmol l^−1^) and copper sulfate pentahydrate (6.67 mmol l^−1^) in water was then added to the reaction followed by sodium ascorbate (208 μl of a 100 mmol l^−1^ solution, final concentration 5.0 mmol l^−1^). The upper phase of the reaction mixture was purged with argon and the reaction was agitated overnight on an orbital shaker (∼30 rotations per minute). The progress of the reaction was evaluated as above.

### Peptide coupling of cyclododecane carboxylic acid and amine-diazirine-alkyne

Cyclododecane carboxylic acid (23 mg, 0.11 mmol) was added to a round bottom flask containing a stir bar under an argon atmosphere. Anhydrous DMF (1.0 ml) was introduced into the flask followed by 28 μl DIPEA and 51.6 mg HATU and the reaction was stirred for 30 min. Then, 2-(3-but-3-yn-1-yl)-3H-diazirin-3-yl)ethan-1-amine (1.50 equiv., 0.165 mmol, 1.00 ml) was added dropwise to the reaction, which was allowed to proceed overnight, with the reaction monitored as above.

### Construction of cyclododecane-diazirine-biotin probe via click chemistry

Cyclododecane-diazirine-alkyne (6.0 mg, 0.018 mmol) was resuspended in 2.0 ml 50:50 tert-butanol:water (concentration in the final reaction: 4.5 mmol l^−1^). Biotin-PEG11-azide (2.0 ml of a 10 mmol l^−1^ solution in 50:50 tert-butanol:water, concentration in the final reaction: 5.0 mmol l^−1^) was added to the alkyne. A freshly prepared, premixed solution of THPTA ligand (33.3 mmol l^−1^) and copper sulfate pentahydrate (6.67 mmol l^−1^) in water (70 μl for a final concentration CuSO_4_⋅5H_2_O=0.10 mmol l^−1^, THPTA=0.50 mmol l^−1^) was added followed by sodium ascorbate (225 μl of a 100 mmol l^−1^ solution, final concentration: 5.0 mmol l^−1^). The upper layer of the reaction was purged with argon and the reaction was agitated overnight on an orbital shaker (∼30 rotations per minute). The progress of the reaction was evaluated as above.

### Probe purification and structural characterization

HPLC purification of synthetic probes was performed using a semi-preparatory Agilent Zorbax SB-C18 column (9.4 mm×25 cm), running a linear gradient 85–100% aqueous acetonitrile over 25 min at a flow rate of 2 ml min^−1^. ^1^H NMR spectra were collected at 700 MHz (Bruker Avance IIIHD spectrometer equipped with 5 mm broadband or inverse detection probe) or 800 MHz (Bruker Avance IIIHD spectrometer equipped with a 3 mm triple resonance cryoprobe). Chemical shifts in ppm (δ) were referenced to deuterated methanol solvent signals (3.31 ppm for ^1^H and 49.1 ppm for ^13^C). High resolution mass spectra (HRMS) were recorded using a ThermoFisher Scientific LTQ Orbitrap XL ETD mass spectrometer by the Systems Mass Spectrometry Core Facility at the Georgia Institute of Technology.

### Animals

Research involving animals was performed in accordance with relevant institutional and national guidelines and regulations. This study and associated procedures were approved by the Institutional Animal Care and Use Committee (IACUC) at Georgia Institute of Technology (A14039) prior to commencement of experiments. Efforts were made to limit the number of animals used to the extent possible. Zebrafish (AB/Tuebingen – wild type) were maintained under standard laboratory conditions (28°C, a 14 h:10 h light:dark cycle; Westerfield, 2000). Embryos were generated by natural spawning as previously described and maintained in Petri dishes at 28°C in 60 μg ml^−1^ of the branded aquarium salt Instant Ocean and 0.1% of the antimicrobial agent Methylene Blue (Kimmel et al., 1995). Fish remained in these Petri dishes until the experiments were conducted. Developmental stages of zebrafish utilized in experiments are reported in days post-fertilization (dpf). Experiments were performed using zebrafish larvae from 6 to 7 dpf. This developmental stage was chosen because early sensory systems are present in these animals, and they have switched to exogenous food and are actively using these sensory systems to make feeding decisions. At the same time, their size at this developmental timepoint makes them ideal for microscopic evaluation as large regions of their bodies may be visualized and pigment development (which is minimal at the 6–7 day timepoint) has not complicated imaging. They also require smaller volumes of water and therefore we could use less of the probes, of which we had limited amounts. Buffered pharmaceutical grade MS-222 was used to anesthetize (50 mg l^−1^) zebrafish larvae during procedures as well as to euthanize them (500 mg l^−1^) at the conclusion of experiments in accordance with institutional and USDA guidelines.

### Exposure of zebrafish larvae to fluorescent formoside-BODIPY and cyclododecane-BODIPY probes

Larval zebrafish (6 dpf) were placed in a 96-well plate containing 100 μl of water per well. Depending on the experiment, there were 2–6 larvae per well/treatment. Formoside-BODIPY probe or cyclododecane-BODIPY probe (1 μl in DMSO) was diluted in 99 μl water and added to the appropriate well to give a final concentration of approximately 65 μmol l^−1^. Larvae were incubated with the probe for 5–10 min and rinsed 3 times in conditioned water taken from the system that directly supplies the zebrafish tanks in the animal facility. This water did not contain Methylene Blue. 2-[4-(Dimethylamino) styryl]-*N*-ethylpyridinium iodide (DASPEI) (final concentration 8 mg ml^−1^) was then added to all wells and larvae were incubated for an additional 30 min prior to being rinsed 3 times and embedded in soft agar on Greiner Bio-One CELLview™ (Monroe, NC, USA) slides for subsequent confocal imaging.

### Exposure of zebrafish larvae to formoside-diazirine-biotin and cyclododecane-diazirine-biotin probes and fluorescent streptavidin

Seven-day post-fertilization (7 dpf) zebrafish larvae were placed into a 96-well plate containing 100 μl H_2_O. Depending on the experiment, each well contained 2–6 larvae. Six treatment scenarios were employed in which larvae were exposed to: (1) DASPEI only, (2) formoside-diazirine-biotin probe and fluorescein streptavidin, (3) cyclododecane-diazirine-biotin probe and fluorescein streptavidin, (4) formoside-diazirine-biotin probe, fluorescein streptavidin and DASPEI, (5) cyclododecane-diazirine-biotin probe, fluorescein streptavidin and DASPEI, or (6) fluorescein streptavidin and DASPEI. DASPEI (or DMSO vehicle in the case of treatments that did not incorporate DASPEI) was added to the larvae first at a final concentration of 8 mg ml^−1^ and incubated for 1 h. At the end of the incubation, larvae were rinsed twice in fish system water. Two microliters of formoside-diazirine-biotin (5 mmol l^−1^ stock in DMSO), cyclododecane-diazirine-biotin (5 mmol l^−1^ stock in DMSO) or DMSO vehicle in 98 μl water (final probe concentration=50 μmol l^−1^) was then added to the appropriate well and incubated for 5 min. At the end of the incubation, larvae were rinsed twice in fish system water. A stock of fluorescein streptavidin in fish system water was prepared (1.5 μl of fluorescein streptavidin/400 μl water) and 100 μl was added to each well and incubated for 2–4 min. Larvae were again rinsed twice in fish system water and were subsequently embedded in soft agar on Greiner Bio-One CELLview™ slides (Monroe, NC, USA) for subsequent confocal imaging.

### Confocal microscopy and image analysis

Images of zebrafish larvae exposed to formoside- and cyclododecane-BODIPY probes and DASPEI, or formoside- and cyclododecane-diazirine-biotin probes, fluorescein steptavidin and DASPEI, were collected at the Georgia Institute of Technology Optical Microscopy Core using a Zeiss 710 NLO laser scanning confocal microscope capable of linear unmixing. Image processing was performed using either Zeiss software or Fiji (Schindelin et al., 2012). Z stacks collected for each image were digitally compiled into maximum intensity projections. Changes to brightness, contrast and color balance, where made, were applied to every pixel in the image. No individual features within images were modified. Multiple images depicting individual zebrafish larvae were grouped together to facilitate comparisons among individuals. Even at the lowest magnification, the entire larva could not fit within the field of view and so images of the head and the rest of the trunk were collected separately, although only the images of the head are shown.

## RESULTS

### Chemical modification of formoside resulted in probes for microscopy

**Fig. 1.**
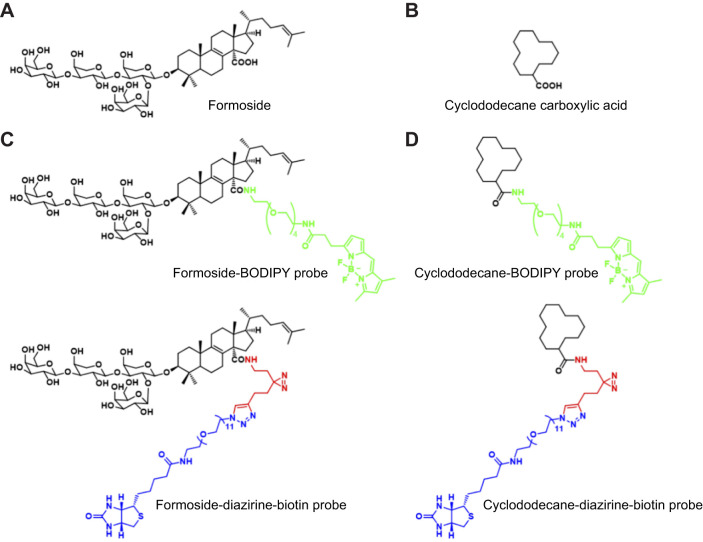
**Structures of probes used in the present study.** Probes were synthesized via a modular approach by functionalizing formoside (A) and the negative control compound, cyclododecane carboxylic acid (B), with probe building blocks (green, bodipy; red, amine-diazirine-alkyne; blue, biotin) that allowed visualization and/or photocrosslinking to cellular targets. Two formoside probes (C) and two cyclododecane probes (D) were thus produced.

Formoside cellular interaction was assessed using chemical probes synthesized in the current study (Fig. 1). Two probes were constructed through the chemical modification of native formoside. In the first, a fluorescent BODIPY moiety was appended to formoside, allowing it to be directly visualized via confocal microscopy. In the second probe, addition of a photocrosslinker to enable covalent bond formation with the probe target, as well as a biotin moiety, allowed for its visualization upon the addition of fluorescein-conjugated streptavidin. Binding specificity of formoside probes was determined by comparison with control probes, consisting of a cyclododecane ring bearing the same moieties used to construct the formoside probes, yielding cyclodecane-BODIPY and cyclododecane-diazirine-biotin. The control cyclododecane moiety was chosen to approximate to the extent possible the size and polarity of native formoside. Synthesis of probes relied on peptide coupling and CuAAC reactions at the site of the carboxylic acid of formoside. This modular, tractable synthetic approach allowed for expedient production of probes with minimal risk of modification to other portions of the molecules. Owing to the large quantities of compound that would be required to conduct behavioral assays and the small synthetic scale employed for synthesis of probes, fish feeding assays were not conducted with synthetic probes.

### Formoside probes localize to zebrafish lips, taste buds and olfactory epithelium

**Fig. 2.**
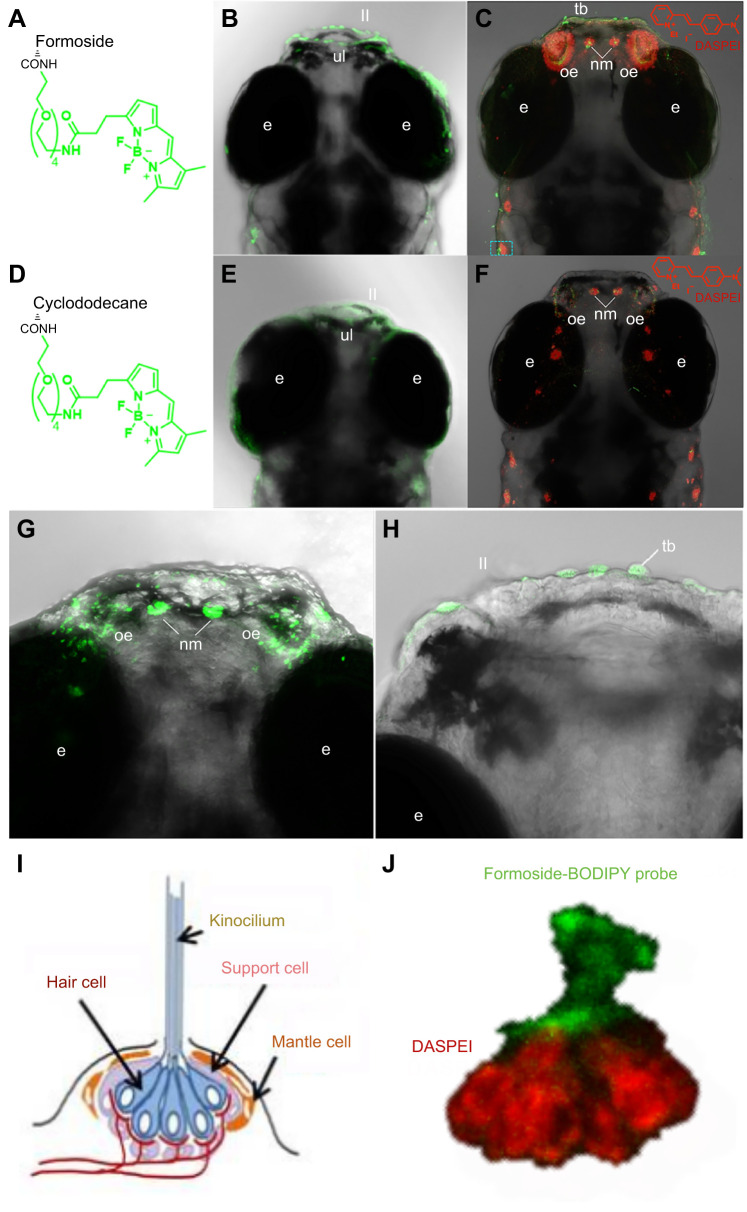
**Confocal microscopy images showing formoside and control probe localization (green) in 6** **dpf larval zebrafish, as well as DASPEI co-localization (red).** The formoside-BODIPY probe (A) localized to putative taste buds on zebrafish lips (B,C,H), the olfactory epithelium (C,G) and neuromast kinocilia (C,G). The negative control cyclododecane-BODIPY probe (D) did not localize to chemosensory or mechanosensory structures (D–F). (I) Schematic of the zebrafish neuromast organization (I) (Mojib et al., 2017, modified from Ghysen and Dambly-Chaudiere, 2004) and (J) confocal microscopy results showing the labeling of different neuromast substructures by formoside-BODIPY and DASPEI. ul, upper lip; ll, lower lip; e, eye; tb, taste bud; oe, olfactory epithelium; nm, neuromast. See Movie 1 for additional images.

**Fig. 3.**
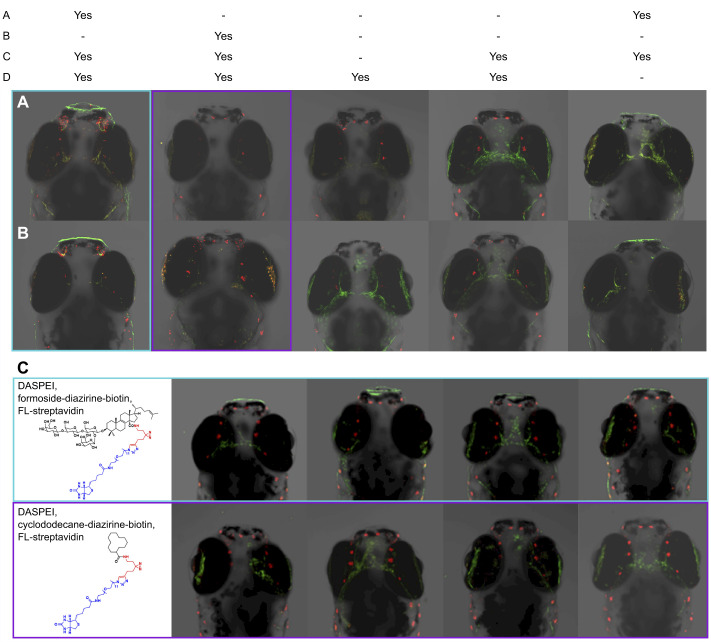
**Confocal microscopy images depicting the localization of biotin-bearing formoside and control probes in 7** **dpf larval zebrafish.** These probes, which are non-fluorescent, were visualized by the addition of fluorescent streptavidin (green). Co-localization with the vital dye DASPEI (red) is also shown. (A,B) Two biological replicates are shown for each treatment: (a) formoside-diazirine-biotin, (b) cyclododecane-diazirine-biotin, (c) fluorescein streptavidin (FL-streptavidin) and (d) DASPEI. (C) Four additional biological replicates are displayed for larvae exposed to either formoside-diazirine-biotin (top row) or cyclododecane-diazirine-biotin (bottom row) along with FL-streptavidin and DASPEI.

Chemosensory structures were sites of formoside probe accumulation in developing zebrafish. Both the formoside-BODIPY probe and the formoside-diazirine-biotin probe paired with fluorescent streptavidin accumulated within the olfactory epithelium (Figs 2 and 3). Visualization of the biotin-bearing probe coupled with fluorescein streptavidin was less obvious owing to higher background or lower signal, perhaps because of differences in the ability of the much larger fluorescent streptavidin to access the formoside-diazirine-biotin probe already bound to its target. Appreciable accumulation of the cyclododecane control probes in the olfactory epithelium was not observed, indicating that localization of the formoside-bearing probes in those locations was due to formoside structural features. Co-localization experiments performed with the vital styryl mitochondrial dye DASPEI showed that this probe, too, localized to the olfactory organ in a time-dependent manner.

The formoside-BODIPY probe was observed to localize to the lips of 6 dpf zebrafish larvae (Fig. 2B). The distribution of the probe was punctate in appearance, accumulating in structures that, based on previous investigation, likely correspond to taste buds (Mojib et al., 2017). Two distinct taste bud morphologies representing the early primordial (Fig. 2H) and mature taste buds (Fig. 2J), respectively, were highlighted by the formoside-BODIPY probe in 6 dpf zebrafish. The formoside-diazirine-biotin probe also displayed localization to the lips of 7 dpf zebrafish, though it was less punctate in appearance than the BODIPY probe (Fig. 3A–C). No colocalization with DASPEI was observed in fish lips. At the early zebrafish developmental stage investigated herein, the sensory barbels are not yet sufficiently formed to investigate whether the formoside probes localize to the taste buds that cover these structures. The labeling patterns observed in larval zebrafish for both probes were consistent and reproducible among biological replicates and experiments.

### Formoside probes accumulate in zebrafish mechanosensory structures

In addition to chemosensory cells, formoside probes were observed to localize to the mechanosensory neuromasts of developing zebrafish (Fig. 2G,J and 3). These structures are responsible for detecting movement and helping fish to orient themselves and capture prey. Identification of neuromasts as the structures to which the formoside probes were binding was confirmed through co-labeling experiments with DASPEI, which is well known to label mechanosensitive hair cells in neuromasts (Harris et al., 2003). Interestingly, formoside probes labeled the stereocilia and kinocilium whereas DASPEI labeled the body of the hair cells (Fig. 2J).

### Free formoside competes with formoside-BODIPY probe for binding to chemosensory and mechanosensory structures

In a preliminary experiment, the addition of free formoside to larval zebrafish incubation experiments with the formoside-BODIPY probe demonstrated that excess native formoside at greater than equimolar concentrations (75 and 100 μmol l^−1^) outcompeted probe binding (at 50 μmol l^−1^), resulting in a loss of fluorescent probe localization to taste buds, olfactory epithelium and neuromasts (Fig. S3).

## DISCUSSION

### Formoside localization to olfactory tissue and taste buds is consistent with its role in chemical defense

The direct tagging of zebrafish cellular structures with a small molecule ligand known to be responsible for aversive behavior in a multitude of fish species (Kubanek et al., 2000) represents new knowledge of the physiology that underlies predator responses to prey chemical defenses (Figs 2 and 3). Although information about the zebrafish physiological response to formoside and its probable dependence on the zebrafish co-receptor RL-TGR was previously gained by studying signal transduction in response to triterpene glycoside exposure of fish tissues (Cohen et al., 2010), the chemical biology probe-based approach described here directly and conclusively demonstrates that this chemical defense molecule localizes to zebrafish chemosensory structures, as well as to mechanoreceptors. The lack of localization to chemosensory and mechanosensory structures by cyclododecane control probes, in conjunction with the attenuation of formoside-BODIPY binding observed in the presence of excess free formoside, supports the conclusion that localization of the probe is directly attributable to the structural features of formoside. In addition, competition between native formoside and the formoside-BODIPY probe suggests that the binding properties of formoside were not substantially altered by its derivatization as a probe and supports the hypothesis that formoside probes and native formoside engage zebrafish receptors in the same way. Therefore, we expected that zebrafish would respond to the formoside probes as they do to formoside, but we cannot be certain as we did not have sufficient quantities of the synthetic probes to conduct behavioral fish feeding experiments.

Based on our observation of formoside localization to the zebrafish olfactory epithelium and taste buds (Figs 2 and 3), it is probable that formoside binds specifically to a receptor or multiple receptors on OSNs and taste receptor cells (as well as neuromasts). RL-TGR, a known chemoreceptor for formoside, has been observed to be expressed in these chemosensory and mechanosensory tissues (Mojib et al., 2017). RL-TGR functions in conjunction with a GPCR (Cohen et al., 2010) and the sensory epithelium of the zebrafish olfactory organ contains five described types of olfactory sensory neurons, each of which expresses combinations of GPCR olfactory receptors, responding to odorants through the regulation of cyclic AMP (Calvo-Ochoa and Byrd-Jacobs, 2019). The resulting electrical signals are processed in the brain, leading to a variety of behavioral responses. Expression of olfactory receptors in zebrafish larvae has been observed to begin 24 to 48 h post-fertilization (Barth et al., 1996; Byrd et al., 1996). Electrophysiological experiments that monitor the responses of single OSNs or taste buds to ligand exposure may be useful to verify that these are in fact the structures to which formoside binds and may furthermore identify the specific OSN subtype targeted by this chemical defense.

In zebrafish, taste buds develop later than the olfactory system, with primordial cells appearing at 3–4 dpf, and taste buds with open receptor areas coinciding with the initiation of feeding at 4–5 dpf (Hansen et al., 2002). Taste buds are also ultimately present on the sensory barbels, which arise later in zebrafish development. Both the mature fish taste buds that were canonical pear- or onion-shaped with a brushlike apical ending, and the early taste bud primordia that were of roundish morphology (Hansen et al., 2002) were highlighted by the formoside-BODIPY probe in 6 dpf zebrafish (Fig. 2). It appears likely that chemoreceptors interacting with formoside are expressed early during taste bud development.

Formoside localization to chemosensory structures is not completely unexpected given its ecological function as a feeding deterrent (Kubanek et al., 2000) and the previously reported expression of RL-TGR (Mojib et al., 2017). However, although formoside binding to chemosensory (and mechanosensory) tissues is physiologically relevant, it is questionable whether the neuromasts and/or olfactory epithelium would encounter formoside in a natural setting. This is because formoside has been shown to be associated primarily with sponge tissues with limited solubility in water (Kubanek et al., 2002). Thus, although our model system, with its increased formoside solubility, has yielded physiologically important insights into the machinery that underpins zebrafish sensation, it also presents ecological caveats that should be borne in mind.

The association of the formoside probes with neuromasts (Fig. 2) is curious. Labeling of the hair bundle (kinocilium/stereocilia) with formoside probes, in contrast to the staining of the main body of the cells within the rosette with DASPEI (Fig. 2J), is intriguing although consistent with previous mapping of RL-TGR gene expression, which revealed that the putative triterpene glycoside co-receptor is present in neuromasts of zebrafish larvae (Mojib et al., 2017). The lack of neuromast tagging by the control probe (Fig. 2), possessing a relatively similar molecular size and polarity profile to the formoside probe, refutes the hypothesis that localization was driven by non-specific interactions between the synthetic probes and the gelatinous cupula that contains the hair bundle.

Taste buds have microvilli whereas neuromasts and some OSNs are ciliated, with the cilia of OSNs and neuromasts sharing the same microtubule configuration (Hara, 2011). OSNs in zebrafish are of five different types: ciliated, microvillus, crypt, kappe and pear neurons (Calvo-Ochoa and Byrd-Jacobs, 2019). One molecular marker, calretinin, exhibits immunoreactivity to most types of OSNs and other olfactory cells in adult zebrafish (Germanà et al., 2007). A recent study suggested that calretinin can be used as a specific marker of ciliated OSNs in olfactory epithelium and light cells of oral taste buds, but this needs to be investigated further (Aragona et al., 2022). In our previous study where we used calretinin to map expression of the formoside-binding receptor RL-TGR, we observed RL-TGR expression to be present in some but not all calretinin-positive cells in olfactory epithelium (Mojib et al., 2017). We also observed RL-TGR expression in oral taste buds that were calretinin-positive. We suggest that perhaps only ciliated OSNs or light cells with one long final microvillus of oral taste buds express formoside-binding RL-TGR. In addition, the neuromast stereocilia express mechano-electric transducer channels at their tips and have been observed to be subject to antagonism by other small molecule natural products (Farris et al., 2004). Whether this is the case with formoside and whether such an action is independent of, or is related to, the mode of formoside binding in OSNs and taste buds merits further investigation.

### The pattern of formoside localization mirrors that of RL-TGR and provides further support for RL-TGR involvement in chemical defense sensing

Localization of formoside probes was highly congruent with previously observed RL-TGR expression in larval zebrafish taste buds, olfactory epithelium and neuromasts (Mojib et al., 2017). These observations provide further support for the involvement of this co-receptor in triterpene glycoside reception. Because the receptor-ligand binding experiments reported herein are premised on having a native, functional receptor, live larvae were used. Therefore, co-localization experiments with formoside and RL-TGR, whose expression would need to be examined via immunohistochemistry, were not possible. Attempts to create a transgenic zebrafish in which RL-TGR incorporates a fluorescent label is a future goal.

Interestingly, RL-TGR may be specific to zebrafish, but formoside-impregnated foods are rejected by fishes that may not have an expressed version of RL-TGR (Kubanek et al., 2000). There are several possible explanations for this phenomenon. It may be that an as yet unidentified RL-TGR homolog exists in these fish species or that the function served by RL-TGR is satisfied in some other manner; alternatively, in these animals, the GPCR responsible for detecting formoside may do so in the absence of a RAMP such as RL-TGR. Extension of the formoside probe-based visualization experiments performed here to other, presumably non-RL-TGR-containing fishes may provide some insight into this idiosyncrasy.

### Conclusion

To our knowledge, these results represent the first visualization of a sponge marine chemical defense interacting with a model predator's sensory tissues, and it is our expectation that future work will ultimately provide mechanistic insight into the process of chemoreception in marine chemical ecology.

## Supplementary Material

10.1242/jexbio.246246_sup1 Supplementary information
