# Supplementary material for: Visualization of the chemical defense molecule formoside binding to sensory structures in a model fish predator
Source: J Exp Biol. 2023 Dec 21;226(24):jeb246246. doi: 10.1242/jeb.246246 (PMC10753513; doi:10.1242/jeb.246246)
Supplement: Supplementary information [file jexbio-226-246246-s1.pdf]

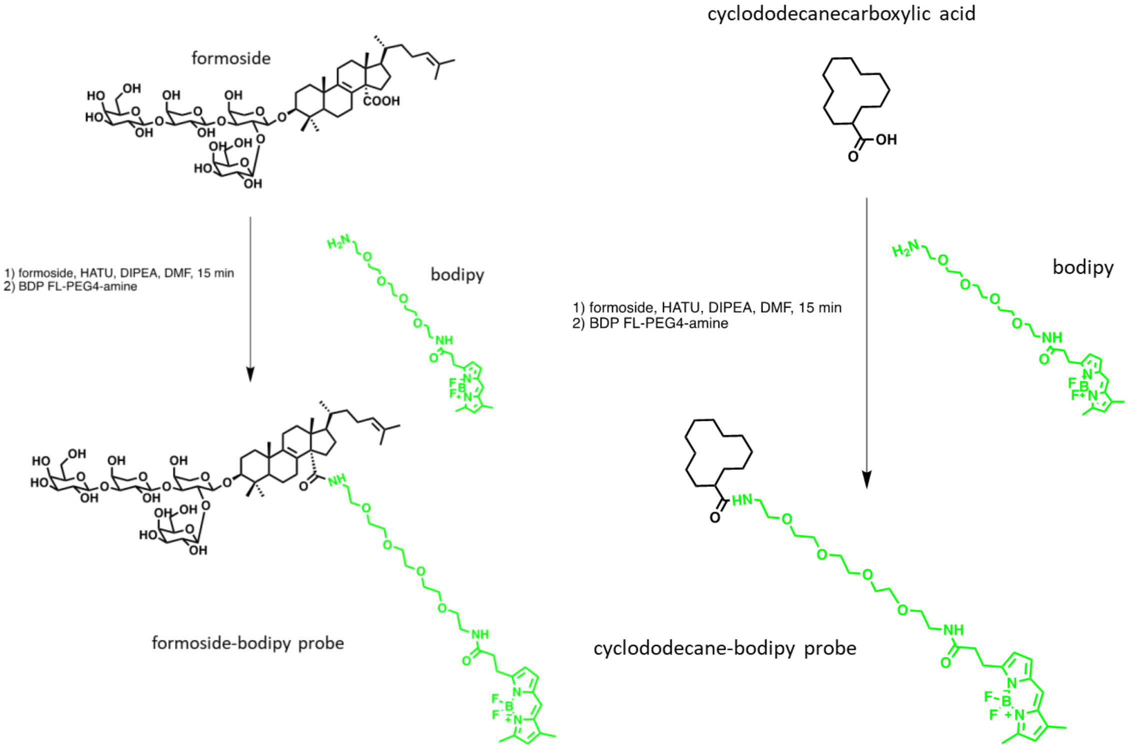

Fig. S1. Synthetic reaction scheme for the formation of (left) formoside- and (right) cyclododecane-BODIPY probes.

Journal of Experimental Biology • Supplementary information

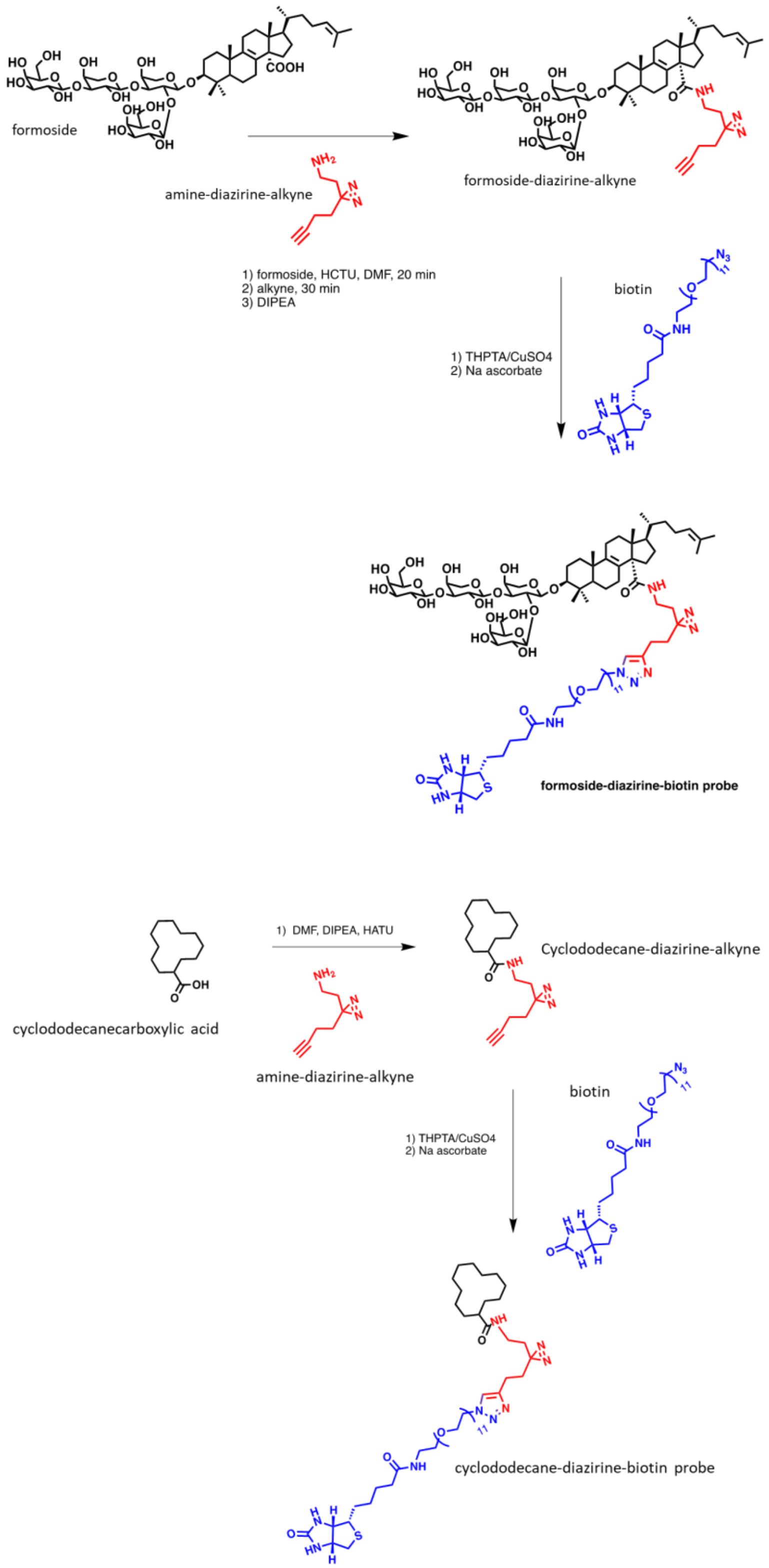

Fig. S2. Synthetic reaction scheme for the formation of (top) formoside- and (bottom) cyclododecane-diazirine-biotin probes.

Journal of Experimental Biology • Supplementary information

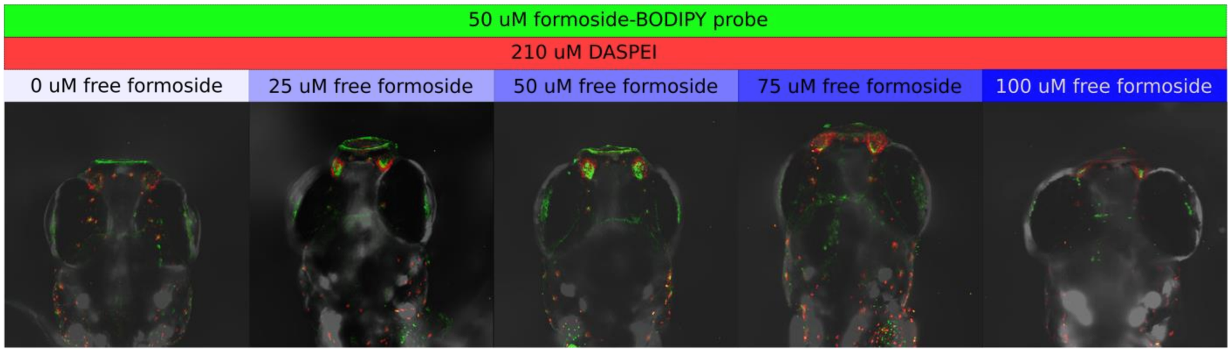

Fig. S3. Preliminary competition experiment between free formoside and the formoside-BODIPY probe. Formoside was added to larval zebrafish (at the concentrations shown above) and after 10 minutes, the probe was added, and the larvae were stained with DASPEI. Each confocal microscope image displays a representative larva for each treatment condition. While the experiment started with a total of 25 fish across all treatments, mortality resulted in a smaller number of larvae being imaged (n=1-5 per treatment).

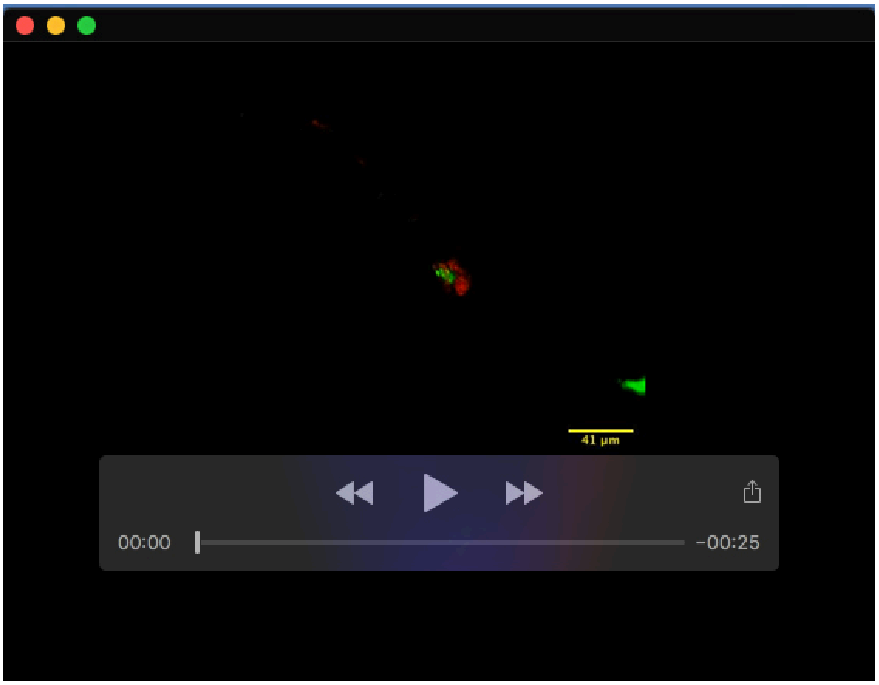

Movie 1. Video animation of confocal Z-stack of zebrafish neuromast labeled with formoside-BODIPY probe and DASPEI (Double click on the icon to play video).
